# The relationship between ocular and oral dryness in a cohort from the 65-year-old population in Norway

**DOI:** 10.1038/s41598-022-13985-6

**Published:** 2022-06-13

**Authors:** Håvard Hynne, Behzod Tashbayev, My Tien Diep, Anne Thea Tveit Sødal, Reza A. Badian, Xiangjun Chen, Xiaoran Lai, Tor P. Utheim, Lene Hystad Hove, Janicke Liaaen Jensen

**Affiliations:** 1 Department of Oral Surgery and Oral Medicine, Faculty of Dentistry, University of Oslo, Oslo, Norway; 2 Department of Cariology and Gerodontology, Faculty of Dentistry, University of Oslo, Oslo, Norway; 3 Department of Medical Biochemistry, Oslo University Hospital, Oslo, Norway; 4 Oslo Centre for Biostatistics and Epidemiology, Faculty of Medicine, University of Oslo, Oslo, Norway; 5 Department of Oral Biology, Faculty of Dentistry, University of Oslo, Oslo, Norway; 6 Department of Plastic and Reconstructive Surgery, Oslo University Hospital, Oslo, Norway

**Keywords:** Eye diseases, Oral diseases, Geriatrics, Dental public health, Gerodontics, Xerostomia, Epidemiology

## Abstract

In the present study, the relationship between dry eyes and dry mouth was explored in 150 65-year-old subjects randomly selected from the general population in Oslo, Norway. The number of drugs, including xerogenic drugs, and current and previous systemic diseases were recorded. Ocular parameters recorded were the McMonnies Dry Eye Questionnaire, the Ocular Surface Disease Index, the Schirmer I Test, tear film break-up time and ocular surface staining. The oral parameters were xerostomia frequency, Summated Xerostomia Inventory, Clinical Oral Dryness Score, and unstimulated and stimulated whole saliva. The participants with current or previous systemic diseases had significantly more ocular and oral symptoms and significantly more oral clinical findings than the participants without a history of disease. Moreover, correlation and factor analyses demonstrated an association between subjective ocular and oral parameters. A significant correlation between the total number of drugs and the presence of ocular and oral symptoms was also noted. When the participants were categorized based on their ocular symptoms, poorer values were found for the oral parameters among the participants more troubled with dry eyes. The results in the present study call for increased awareness and an interdisciplinary approach in matters related to dry eyes and dry mouth.

## Introduction

Symptoms of dry eyes and dry mouth are common in the elderly population^1^. Dry eyes and dry mouth are separately reported in up to 30% of the general population above 65 years of age, being more common among women than in men^1–5^.

Dry eye disease (DED) is a major public health concern impacting general quality of life^6^. DED is defined by The Tear Film & Ocular Surface Society Dry Eye Workshop II report as: *“A multifactorial disease of the ocular surface characterized by a loss of homeostasis of the tear film, and accompanied by ocular symptoms, in which tear film instability and hyperosmolarity, ocular surface inflammation and damage, and neurosensory abnormalities play etiological roles.”*^7^. The symptoms can vary, but in general, DED presents with watering, itching, burning sensation of the eyes, ocular discomfort, and pain^8,9^. The common risk factors for DED include age^2^ and the use of medications^9^. More than 60% of DED cases in the elderly population have been attributed to medications^10^. Systemic conditions such as Sjögren’s syndrome, and diabetes mellitus have also been identified as risk factors for DED^6^.

Unlike DED, there is no common definition of dry mouth disease. Dry mouth includes both xerostomia and hyposalivation. Subjective feeling of dry mouth is defined as xerostomia, while objective demonstration of reduced salivary secretion is defined as hyposalivation^11^. Xerostomia and hyposalivation do not necessarily correlate^12^. Reduced salivary secretion may lead to deteriorated oral health, including caries, *Candida* infection, distorted taste, and even pronounced difficulties with speech and swallowing^11,13^. The etiology of dry mouth is multi-faceted. Common risk factors include medications such as antidepressants, anticholinergics, antispasmodics, antihypertensives, antihistamines, sedatives, and diuretics^14^. Known systemic conditions that may lead to hyposalivation include Sjögren’s syndrome, diabetes mellitus, and Parkinson’s disease^14^. Head and neck malignancies treated with irradiation are another well-known risk factor^14^. In addition, dehydration is associated with hyposalivation, and affects 20–30% of older adults^15^.

DED and dry mouth have been studied extensively as standalone conditions. A few studies have reported the association between DED and xerostomia in patient populations such as Sjögren’s syndrome^16,17^, connective tissue disorders^18^, diabetes mellitus^19^, and psychiatric disorders^20^. However, there is a paucity of data on the association between DED and dry mouth in the general population. If DED and dry mouth are to be associated in the general population, it may have an impact on treatment strategies, and in turn enhance interdisciplinary referral practice.

Our research group has previously studied patients with primary Sjögren’s syndrome in detail^21–26^, and we recently initiated studies on cancer patients after head and neck radiation. Our published results show correlations between the ocular and oral parameters in these groups of patients^23,27,28^. These findings have encouraged us to investigate the possible relationship between ocular and oral parameters in the general population, more specifically, in the young elderly. To our knowledge, the relationship between subjective and objective ocular and oral parameters has not been investigated in cross-sectional studies of the young elderly.

The aim of the present study was to explore the relationship between several parameters of dry eyes and dry mouth in a cohort from the 65-year-old population.

## Participants and methods

This cross-sectional study is part of a larger project focusing on oral health in the 65-year-old population in Oslo, Norway (the OM65-study)^29^ and was carried out as a collaboration between the Faculty of Dentistry, University of Oslo; and the Norwegian Dry Eye Clinic. The Norwegian Regional Committee for Medical and Health Research Ethics approved the study protocol (REK 2018/1383).

In the OM65-study, a random sample of eligible individuals was drawn from the Norwegian tax registry. The eligibility criteria were: Born in 1954, and residing in Oslo, Norway. The names and addresses of the selected people were obtained, and invitation letters were sent out. Within two weeks, those invited were called by phone and asked if they wanted to participate in the study. All invited individuals were included and examined upon the acceptance from the participant. The participants were recruited consecutively. The study was performed in compliance with the tenets of the Declaration of Helsinki. Written informed consent was obtained from all participants prior to participation in the study.

### Participants

**Figure 1:**
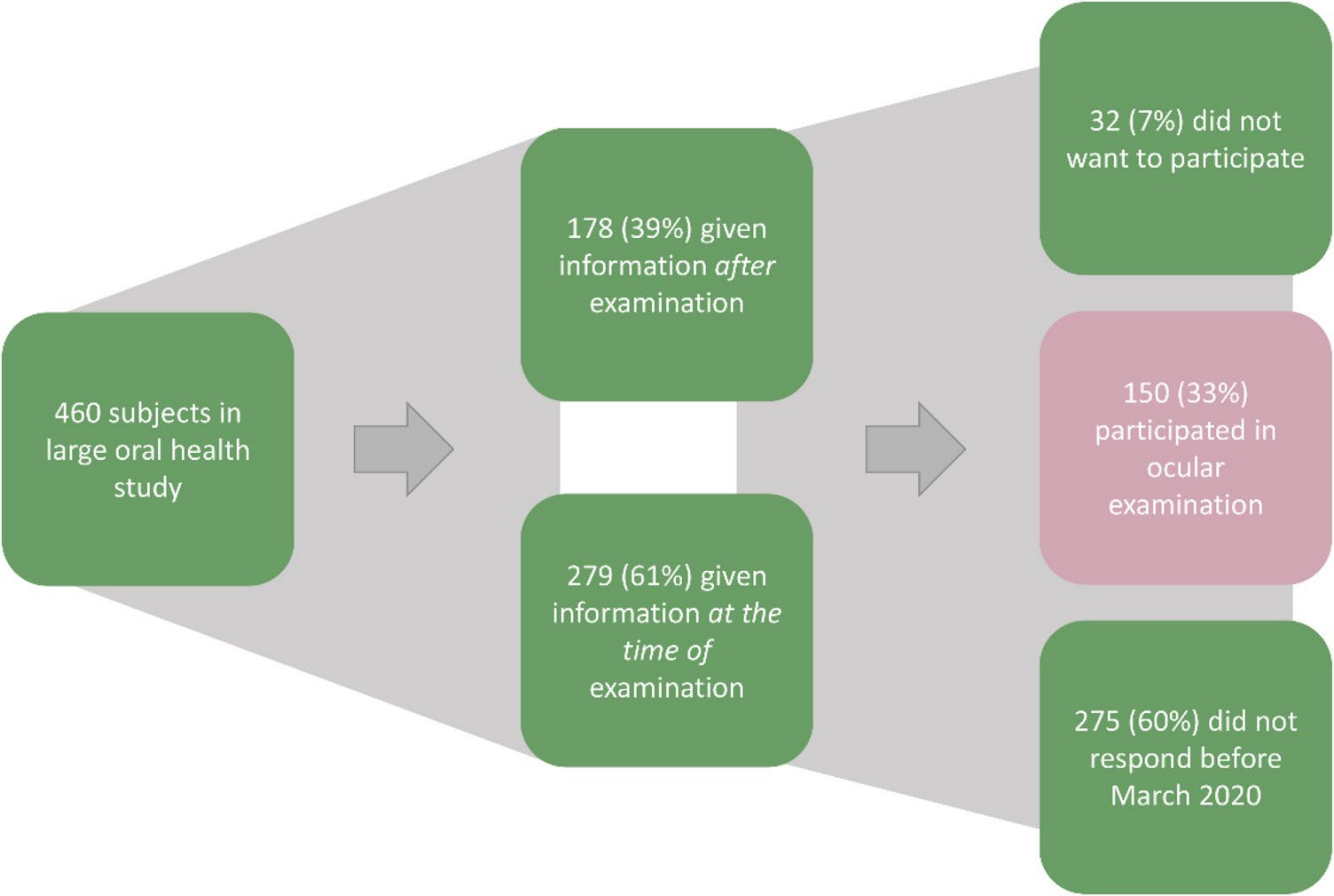
Flow diagram illustrating the recruitment process.

A total of 460 participants attended the examination in the OM65-study (response rate: 58%), and all participants were invited to participate in a sub-study on ocular health. The participants from the OM65-study were given written information about the sub-study on the day of the oral examination or thereafter by mail. Our aim was to include as many as possible from the main study; however, due to the coronavirus 2019 (Covid-19) pandemic, we decided to stop inclusion in March 2020. At that point, 150 participants had been enrolled in the sub-study. The flow diagram shows the recruitment process (Fig. 1).

### Methods

#### Examination of ocular health

##### Patient-reported outcomes

All participants underwent subjective and objective dry eye examinations at the Norwegian Dry Eye Clinic. The examinations were performed from June 2019 to February 2020 between 4 p.m. and 7 p.m. by two experienced ophthalmologists.

Prior to the clinical examination, subjective evaluation of DED was performed using two questionnaires: The McMonnies Dry Eye Questionnaire (MDEQ)^30^ and the Ocular Surface Disease Index (OSDI)^31^. The MDEQ is one of the most widely used patient-reported screening instruments for DED. The questionnaire helps to detect DED and to identify patients at risk of developing this disease. The MDEQ includes questions regarding both risk factors and demographic factors, and the total score ranges 0–45, where higher scores indicate greater severity of symptoms. The MDEQ is best utilized as a screening test for discriminating people with dry eyes from the general population, and not as a grading tool of DED severity^32^.

As the MDEQ questionnaire is used mainly as a screening method for dry eyes and the present cohort was recruited from the general population, we attempted to maximize the sensitivity to avoid missed diagnosis. Accordingly, the cut-off value for the MDEQ was set to 10.5^33^. The OSDI questionnaire is a tool for measuring the severity of ocular surface symptoms related to chronic DED, and their effect on the patient’s ability to function. The OSDI covers environmental triggers, and visual performances that are not included in the MDEQ. The OSDI score ranges 0–100, where higher scores indicate greater severity of symptoms. A score of 0–12 represents a normal state, 13–22 indicates mild DED, 23–32 indicates moderate DED, while 33–100 indicates severe DED^31,34^. In addition, a detailed description of what medications the participants were currently taking was noted. All medications noted were classified according to the Anatomical Therapeutic Chemical Classification System, and their possible xerogenic effect was classified based on already published literature and the Summary of Product Characteristics^35,36^.

##### Clinical examination

Following completion of the dry eye questionnaires, all participants underwent an ocular examination using slit lamp biomicroscopy. The protocol and order of the examinations were identical for all participants. Tear film stability was assessed by examining the tear film breakup time (TFBUT). For the TFBUT, the tear film was evaluated by staining with fluorescein and measuring the interval that elapsed between a blink and the first break in the tear film^37^. The TFBUT was measured after 5 µL 2% fluorescein sodium had been applied to the lower palpebral conjunctiva using a micropipette, and an average of three measurements was recorded. Values of < 10 s were considered abnormal^38^.

Grading of ocular surface staining (OSS) was performed according to the Oxford grading scheme using fluorescein, and yellow barrier filter on the biomicroscope. Positive staining indicates damaged epithelial cells of the cornea and the conjunctiva, and OSS is therefore an important parameter in DED diagnostics^37^. The Oxford Grading Scheme categorizes conjunctival and corneal staining into 6 grades: 0—absent, I—minimal, II—mild, III—moderate, IV—marked, and V—severe^39^.

Aqueous tear production was measured using the Schirmer I test without anesthesia. The Schirmer I test was performed by placing the Schirmer paper strip at the temporal one-third of the lower lid margin. The length of the wetting of the Schirmer strip in millimeters after 5 min was recorded^37^. The cut-off values of the Schirmer I test vary^40^, but a value of < 10 mm/5 min is often considered abnormal and was used in the present study^37^.

#### Examination of dry mouth

The participants’ oral health was examined prior to the ocular examination. The examinations were conducted at The Research Clinic at the Faculty of Dentistry, University of Oslo, as part of the OM65-study. All participants were instructed to refrain from eating, drinking, and smoking 1 h prior to their appointment. The examinations were performed from February 2019 to December 2019 between 8 a.m. and 3 p.m. by two experienced dentists. All participants were examined with the two dentists present.

##### Patient-reported outcomes

The participants were asked to respond to an electronically self-administered questionnaire prior to their appointment. The general xerostomia question was interpreted as the xerostomia frequency: “How often does your mouth feel dry?” with the response options: Never = 1, occasionally = 2, frequently = 3, and always = 4^41^. For the general xerostomia question, case definition for dry mouth was based on a response of ≥ 3^41^. The participants were then asked to respond to the five statements that form the Summated Xerostomia Inventory-Dutch version (SXI)^42^. The SXI is a shortened version of the Xerostomia Inventory^43^ questionnaire used to determine the severity of xerostomia. The SXI sum score ranges 5–15, where the maximum sum score indicates extremely severe problems related to dry mouth. There is no established cut-off value for SXI. Here, case definition for dry mouth was based on a summated response of > 10. To achieve a score > 10 respondents must obtain the highest score on at least one item.

##### Clinical examination

An objective score for oral dryness was obtained using the Clinical Oral Dryness Score (CODS)^44^. The CODS is determined from 10 different features of oral dryness, and each positive feature scores 1 point, with higher scores indicating more severe oral dryness.

Unstimulated whole saliva (UWS) and chewing-stimulated whole saliva (SWS) were collected, as previously described^29^. In brief, subjects were asked not to eat, drink or smoke at least 1 h before saliva collection. For UWS, the participants were asked to avoid swallowing and to spit regularly into a plastic cup for 5 min. For SWS, the participants were asked to chew on a paraffin block (Paraffin Pellets, Ivoclar Vivadent, Shaen, Lichtenstein), while saliva was collected for 5 min. The saliva samples were weighed, and the salivary secretion rates were calculated as mL/min, using 1 g saliva = 1 mL saliva. Values ≤ 0.1 mL/min were considered pathological for UWS, and values ≤ 0.7 mL/min were considered pathological for SWS^45^.

### Statistical analyses

The statistical analyses were performed with the commercial software SPSS for Windows, version 26 (IBM, Chicago, IL) and RStudio, version 1.3.959 (RStudio Team, 2020). Missing values were replaced with the mean value of all responses for continuous variables, and the mode for categorical variables (Table 2 presents the number of missing cases for all parameters). The normality of variables was verified by the Shapiro–Wilk tests. The means of all data for ocular and oral measurements in the male and female participants were compared. The independent T-test was used in for comparing parameters with normal distribution, while the Mann–Whitney U test was used for parameters with non-normal distribution. One-way ANOVA was used in the intergroup comparison of parameters. Correlations between variables were determined using Spearman’s rho correlation analyses (r = 0–0.19, very weak; r = 0.2–0.39, weak; r = 0.40–0.59, moderate; r = 0.6–0.79, strong; r = 0.8–1, very strong).

Exploratory factor analysis was performed to characterize the participants according to both the DED and the dry mouth datasets. In factor analysis, multiple observed variables are described by their relationship to an unobserved (not directly measured) variable based on the similar patterns of responses or findings. Based on the values from the correlation calculation, we removed the variables OSDI, SWS, xerostomia frequency, and number of xerogenic drugs prior to the analysis to avoid multicollinearity. The Kaiser–Meyer–Olkin Measure was calculated to test the degree of common variance, and Bartlett’s Test of Sphericity was significant, hence the sample was found acceptable for factor analysis. The use of two factors in the factor analysis was calculated to be sufficient. Having selected the number of factors for the model, we then proceeded to examine the loading values to determine the variable with the most influence on each factor. The loading value is the correlation coefficient for the variable and factor, and a loading value close to -1 or 1 indicates that the factor strongly influences the variable. Values from the component transformation matrix were inspected, and varimax rotation was chosen. Varimax rotation is a statistical technique that helps in identifying the factor on which the data load. This is done by removing the middle ground, and maximizing the variance shared among variabels^46^. The following packages were used during the factor analysis and in the construction of the correlation plot: psych (v. 2.0.8; Revelle, 2020), GPArotation (v. 2014.11.1; Coen, Bernaards, and Jennrich, 2005), corrplot (v. 0.84; Wei and Simko, 2017), ggplot2 (v. 3.2; Wickham, 2016), cowplot (v. 1.0.0; Wilke, 2019), and PerformanceAnalytics (v. 2.0.4; Peterson et al., 2020) for R programming language.

The reported results are presented as the mean ± standard deviation (SD). A p-value of < 0.05 was chosen as significant, and Bonferroni correction was performed when multiple hypotheses were tested.

## Results

Demographic characteristics and medical history of the 150 participants are listed in Table 1. In the present cohort, there were more women than men, 90% of the participants were born in Norway, 96% had secondary or higher education, 61% had no current or previous diseases, and 28% were taking no drugs at the time of examination. A detailed overview on the generic names of the drugs, ATC-codes, number of participants taking the drug, and potential xerogenic effect is included in Supplementary Table S1.

**Table 1:** Demographic characteristics of participants (n = 150).

	Number of participants
**Sex**	
Male	68 (45%)
Female	82 (55%)
**Ethnicity**	
West-European	140 (93%)
Other	10 (7%)
**Education**	
Basic	6 (4%)
Secondary	44 (30%)
Higher	100 (66%)
**Previous diseases**	
Diseases of the circulatory system	16 (10%)
Cancer	21 (14%)
Others	3 (2%)
No previous disease	111 (74%)
**Current diseases**	
Diseases of the respiratory system	15 (10%)
Diseases of the musculoskeletal system/connective tissue	28 (19%)
Cancer	6 (4%)
Diseases of the circulatory system	48 (32%)
Endocrine, nutritional and metabolic diseases	10 (7%)
Others	36 (24%)
No current disease	118 (78%)
**Number of drugs**	
≥ 5 drugs	28 (19%)
< 5 drugs	122 (81%)

### Ocular and oral parameters

Ocular and oral parameters, and number of drugs taken are presented in Table 2. There was a large range in both ocular and oral parameters.

**Table 2:** Mean values, SD, minimum, maximum, and missing values for ocular, oral and drug parameters.

	Ocular parameters					Oral parameters					Number of drugs	
	MDEQ	OSDI	ST	TFBUT	OSS	XF	SXI	CODS	UWS	SWS	ND	NXD
n	148	149	146	139	150	150	150	150	150	148	150	150
Mean	6.3	8.3	12.4	9.0	0.8	1.6	6.7	2.0	0.4	1.9	2.5	0.7
± SD	4.0	11.3	8.6	6.2	1.2	0.7	1.7	1.3	0.3	0.9	2.8	1.2
Minimum	0	0	2	0	0	1	5	0	0.04	0.24	0	0
Maximum	20	64	36	36	5	4	15	6	1.3	4.9	13	8
Missing	2	1	4	11	0	0	0	0	0	2	0	0

*MDEQ* McMonnies Dry Eye Questionnaire, *OSDI* Ocular Surface Disease Index, *ST* Schirmer I Test (mm/5 min), *TFBUT* tear film break-up time, *OSS* ocular surface staining, *XF* xerostomia frequency, *SXI* summated xerostomia inventory, *CODS* clinical oral dryness score, *UWS* unstimulated whole saliva (mL/min), *SWS* stimulated whole saliva (mL/min), *ND* number of drugs, *NXD* number of xerogenic drugs.

Table 3 shows the number of subjects who had pathological levels of ocular and oral variables in the cohort of 150 subjects. Dry eyes and dry mouth coexisted in 4% of the subjects investigated based on OSDI > 12 and XF ≥ 3.

**Table 3:** Number of subjects with pathological levels of ocular and oral variables.

		n
Ocular	OSDI > 12	41
	MDEQ > 10.5	24
	TFBUT ≤ 10	96
	TFBUT ≤ 5	59
	ST ≤ 10	73
	OSS ≥ 1	68
Oral	SXI > 10	5
	XF ≥ 3	12
	UWS ≤ 0.1	12
	SWS ≤ 0.7	7

*OSDI* ocular surface disease index, *MDEQ* McMonnies Dry Eye Questionnaire, *TFBUT* tear film break-up time, *ST* Schirmer I Test (mm/5 min), *OSS* ocular surface staining, *SXI* summated xerostomia inventory, *XF* xerostomia frequency, *UWS* unstimulated whole saliva (mL/min), *SWS* stimulated whole saliva (mL/min).

When the cohort was stratified based on current or previous systemic disease versus no current or previous systemic disease (Table 1), there were significant differences in the subjective parameters MDEQ (7.0 ± 4.2 vs. 5.1 ± 3.5, p = 0.008), SXI (7.0 ± 1.9 vs. 6.1 ± 1.1, p = 0.002) and XF (1.8 ± 0.8 vs. 1.4 ± 0.6, p = 0.001), and in the objective parameter CODS (2.2 ± 1.4 vs. 1.5 ± 1.2, p = 0.003).

### Correlations between ocular and oral findings

**Figure 2:**
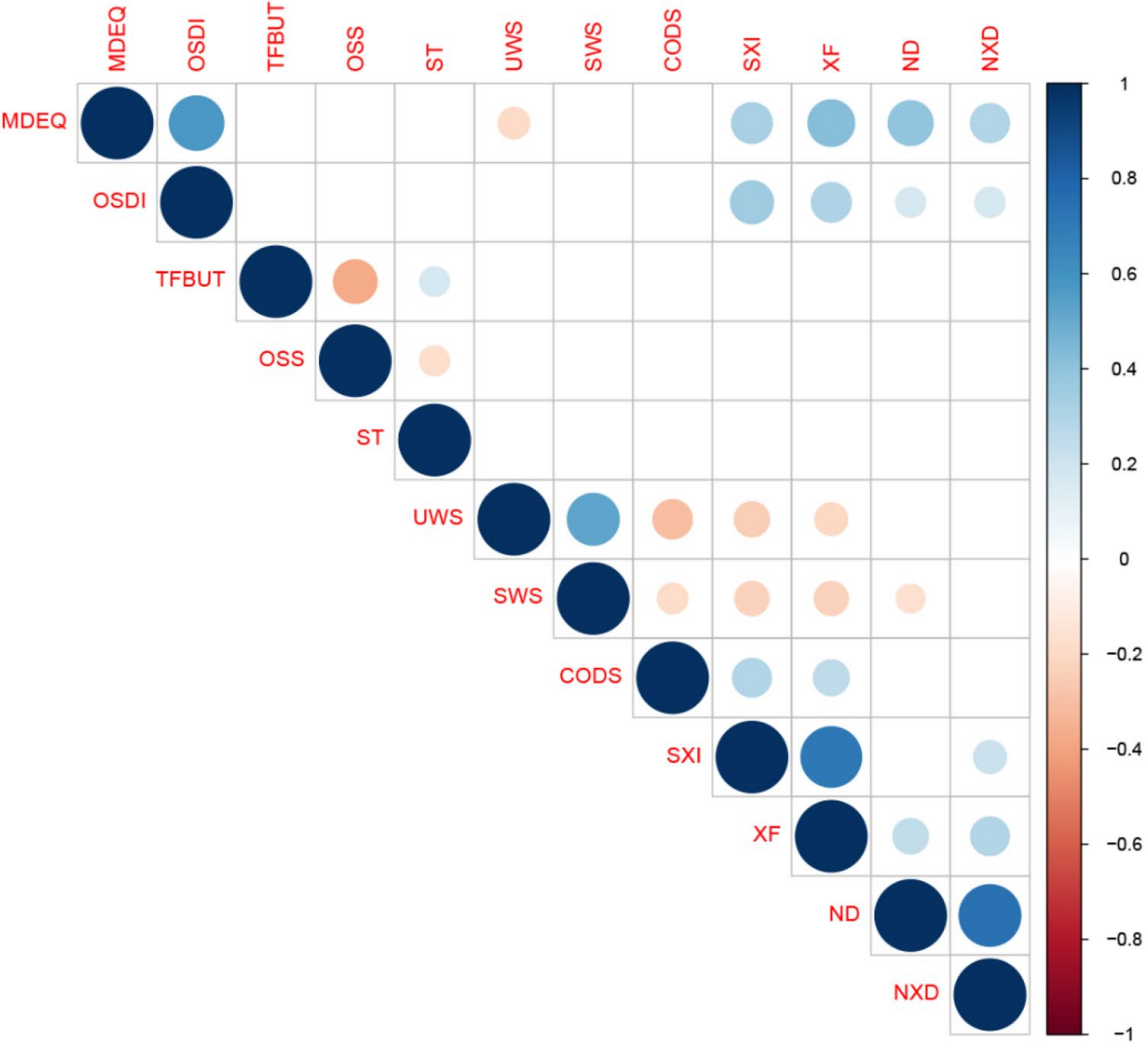
Significant correlations when comparing patient-reported and clinical ocular and oral findings (p < 0.05). Ocular parameters: *MDEQ* McMonnies Dry Eye questionnaire, *OSDI* Ocular Surface Index questionnaire, *TFBUT* tear film break up time (s), *OSS* ocular surface staining, *ST* Schirmer I test (mm/5 min). Oral parameters: *UWS* unstimulated whole saliva (ml/min), *SWS* stimulated whole saliva(ml/min), *CODS* Clinical Oral Dryness Score, *SXI* summated xerostomia inventory, *XF* oral dryness frequency, *ND* number of drugs, *NXD* number of xerogenic drugs.

Figure 2 shows all significant correlations between the subjective and objective ocular and oral findings. The following significant correlations were found after performing Bonferroni correction (p < 0.004): the MDEQ and OSDI showed a weak positive correlation to SXI (r = 0.36, p < 0.001 and r = 0.36, p < 0.001, respectively). The MDEQ and OSDI showed a moderate positive correlation (r = 0.42, p < 0.001) and a weak positive correlation (r = 0.28, p < 0.001), respectively, against xerostomia frequency. The number of drugs and the number of xerogenic drugs showed a weak positive correlation to the MDEQ (r = 0.39, p < 0.001, and r = 0.30, p < 0.001, respectively) and xerostomia frequency (r = 0.25, p < 0.001, and r = 0.25, p < 0.001, respectively). Additionally, the number of xerogenic drugs showed a weak positive correlation to SXI (r = 0.25, p < 0.001).

To explore the relationships between ocular symptoms and oral parameters, subgroups of the ocular parameters OSDI and MDEQ were calculated. The subgroups were formed based on accepted cut-off values for the OSDI and MDEQ^33,34^. Table 4 shows that patients with higher MDEQ values had significantly poorer objective oral findings (UWS and CODS) as well as worse subjective oral findings (xerostomia frequency). Based on OSDI values, significant differences were limited to subjective oral parameters (poorer SXI and higher xerostomia frequency scores). Similar calculations were performed using groups stratified according to ST and TFBUT values. However, no statistical significances were found.

**Table 4:** Comparison of oral parameters between subgroups of *OSDI* Ocular Surface Index questionnaire and *MDEQ* McMonnies Dry Eye questionnaire respectively.

	n	UWS		SWS		CODS		SXI		XF	
		Mean	SD	Mean	SD	Mean	SD	Mean	SD	Mean	SD
OSDI (0–12)	107	0.4	0.3	1.9	0.9	2	1.3	**6.4**^**1**^	1.5	**1.5**^**2&3**^	0.6
OSDI (13–22)	22	0.3	1.8	1.7	0.6	1.6	1.4	7.3	2	**1.9**^**2**^	0.7
OSDI (23–32)	13	0.3	0.2	1.9	1	2	1.1	7	2	1.7	0.8
OSDI (33–100)	6	0.3	0.2	1.6	0.5	3	1.4	**8.5**^**1**^	2.1	**2.3**^**3**^	1
MDEQ (0–10.5)	123	**0.4**^**4**^	0.2	1.9	0.9	**1.9**^**5**^	1.3	6.5	1.5	**1.5**^**6**^	0.6
MDEQ (> 10.5)	24	**0.3**^**4**^	0.2	1.6	0.8	**2.5**^**5**^	1.4	7.4	2.4	**2.1**^**6**^	0.9

ANOVA with Bonferroni Post Hoc test.

Bold represents level of significance: p < 0.05.

^1^*SXI* Summated Xerostomia Inventory, OSDI 0–12 vs. OSDI 33–100, p = 0.016.

^2^*XF* xerostomia frequency, OSDI 0–12 vs. OSDI 13–22, p = 0.032.

^3^XF, OSDI 0–12 vs. OSDI 33–100, p = 0.023 Mann–Whitney U test of relationship MDEQ ≤ 10.5 vs. MDEQ > 10.5 and oral parameters.

^4^*UWS* unstimulated whole saliva (ml/min), p = 0.04.

^5^*CODS* Clinical Oral Dryness score, p = 0.032.

^6^XF, p = 0.002.

When only comparing the relationship between pathological values of oral and ocular variables (Table 5), significant correlations between ocular and oral subjective parameters were detected. Additionally, a significant correlation between reduced tear production (Schirmer test) and xerostomia frequency was obtained.

**Table 5:** Correlation between pathological levels of ocular and oral parameters.

Ocular	Oral				
	SXI > 10	XF > 3	UWS ≤ 0.1	SWS ≤ 0.7	ND ≥ 5
**OSDI > 12**					
r	0.22	0.15	0.02	− 0.05	0.17
p-value	**0.01**	0.07	0.84	0.52	**0.04**
**MDEQ > 10.5**					
r	0.22	0.21	− 0.14	− 0.13	0.30
p-value	**0.01**	**0.01**	0.09	0.11	**0.01**
**TFBUT ≤ 10**					
r	− 0.05	− 0.08	0.09	− 0.04	0.03
p-value	0.59	0.34	0.27	0.68	0.77
**ST ≤ 10**					
r	0.06	0.18	0.13	0.10	− 0.05
p-value	0.52	**0.03**	0.13	0.24	0.55
**OSS ≥ 1**					
r	− 0.20	0.18	− 0.08	0.02	0.05
p-value	0.81	**0.03**	0.35	0.84	0.59
**ND ≥ 5**					
r	0.01	0.17	− 0.05	− 0.20	
p-value	0.94	**0.03**	0.56	**0.02**	

*OSDI* ocular surface disease index, *MDEQ* McMonnies Dry Eye Questionnaire, *TFBUT* tear film break-up time, *ST* Schirmer I Test (mm/5 min), *OSS* ocular surface staining, *ND* number of drugs, *SXI* summated xerostomia inventory, *XF* xerostomia frequency, *UWS* unstimulated whole saliva (mL/min), *SWS* stimulated whole saliva (mL/min).

Bold values represent level of significance: p < 0.05.

**Figure 3:**
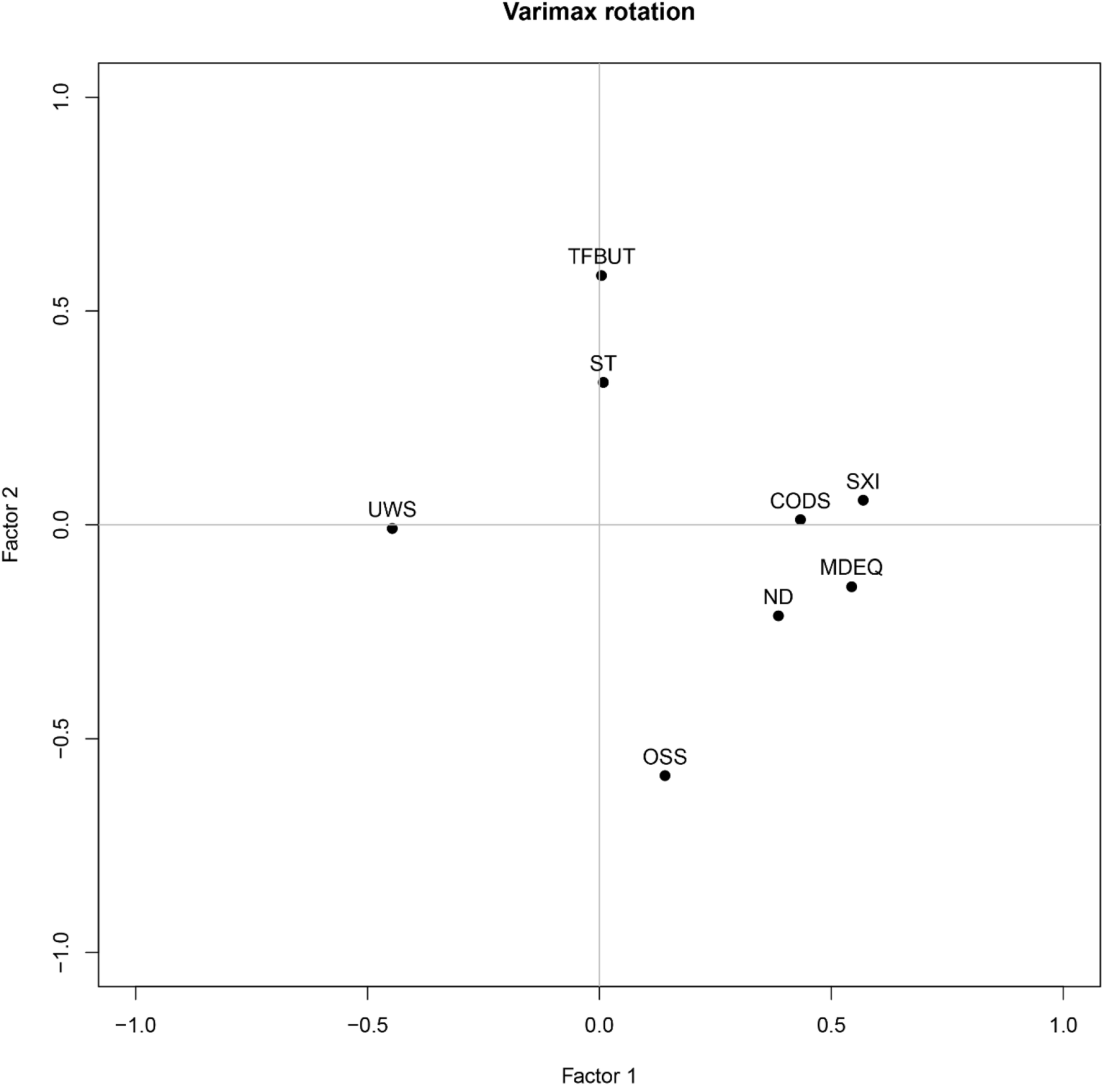
Loading pattern of the influences of the two factors on DED and dry mouth variables after varimax rotation. *MDEQ* McMonnies dry eye questionnaire, *OSDI* Ocular Surface Index questionnaire, *TFBUT* tear film breakup time (s), *OSS* ocular surface staining, *ST* Schirmer I test (mm/5 min). Oral parameters —*UWS* unstimulated whole saliva (mL/min), *SWS* stimulated whole saliva (mL/min), *CODS* clinical oral dryness score, *SXI* Summated Xerostomia Inventory, *ND* number of drugs.

Exploratory factor analysis was performed to characterize the participants according to both DED and dry mouth datasets. For practical and statistical reasons, two factors were chosen as they yield the simplest model with the greatest explanatory power. Figure 3 illustrates the loading pattern of the influences of the two factors on DED and dry mouth variables after varimax rotation.

Figure 3 shows that the variables MDEQ (loading value = 0.55), number of drugs (loading value = 0.39), CODS (loading value = 0.43) and SXI (loading value = 0.57) had the largest positive impact on Factor 1, while UWS (loading value = -0.45) had the largest negative impact. This means that Factor 1 mostly describes oral parameters, but is also influenced by the ocular parameter MDEQ and the number of drugs. For Factor 2, TFBUT (loading value = 0.58) and ST (loading value = 0.33) had the largest positive impact, and OSS (loading value = -0.59) had the largest negative impact. This means that Factor 2 mostly concerns the ocular parameters.

## Discussion

The main finding in the present study was the demonstration of a significant positive correlation between ocular and oral symptoms in the young elderly population. We also revealed that participants with current or previous systemic diseases had more ocular and oral symptoms, and more oral objective findings. Moreover, there was a significant correlation between ocular and oral symptoms and the number of drugs/xerogenic drugs.

When comparing the group with “current or previous systemic disease” with the “no current or previous systemic disease” group, we found significant differences between multiple parameters. The participants with current or previous systemic disease had more symptoms of DED, clinical signs of oral dryness, represented by CODS, and xerostomia compared to subjects with no current or previous systemic disease. These findings are in line with previous studies stating that systemic conditions are important in the etiopathogenesis for both DED and dry mouth^1,14,18,19,47–50^.

We found significant associations between oral and ocular symptoms. However, the associations were only weak or moderate. This indicates that in the general population of the young elderly, those with symptoms of dry eyes may also have symptoms of dry mouth. The association between the subjective ocular parameters and xerostomia frequency also indicates that participants with more subjective problems related to dry eyes had increased frequency of xerostomia. Furthermore, the results showed that dry eyes and dry mouth were present in 4% of the participants when the case definition for dry eyes and dry mouth were set to OSDI > 12 and XF ≥ 3. However, if the case definition for dry mouth was set to also include participants reporting dry mouth “occasionally”, the coexistence increased to 20%. This illustrates the need for a consensus on what tools for patient-reported measurements should be used in research and clinical practice when defining dry mouth and could be a goal for future research.

Exploration of the correlation between the number of drugs/number of xerogenic drugs and the ocular and oral variables, revealed significant associations between the subjective parameters. Systemic medications have been reported to be a major factor in causing both dry eyes and dry mouth^1–4,6,10,11,13,14,19,36,48,51,52^. In contrast, we did not find an association between the number of drugs/number of xerogenic drugs and objective variables when investigating the group as a whole. A possible explanation for this lack of association may be related to the composition of tears and saliva. Jager et al. reported that patients with medication-related xerostomia often have a normal salivary flow rate but reduced protein concentration in the saliva^53^. To our knowledge, similar results for tear production have not been reported, and should be investigated in future studies.

Analysis of variance (ANOVA) showed a statistical significant relationship between some of the oral clinical parameters and the severity of DED. When the participants were categorized based on their ocular symptoms, poorer values were found for the oral parameters among the participants more troubled with DED. When investigating the correlation between pathological ocular parameters and pathological oral parameters, significant correlations appeared between subjective ocular and subjective oral parameters and between the tear production and the frequency of dry mouth. Interestingly, we also found a significant correlation between pathological values for stimulated salivary secretion and taking more than five drugs. Evidently, such correlations were masked when analyzing the whole cohort. Due to the fact that this cohort was recruited from the general population, relatively few participants reported severe problems related to DED and dry mouth, and further correlation analysis was not possible. Still, both the ANOVA and the correlation analysis demonstrated additional relationships between ocular and oral parameters when the most affected subjects from each group were included. Further exploration of this relationship could be a goal for future research.

The factor analysis, aimed at achieving a broader descriptive statement about the study cohort, showed that one group of variables described ocular parameters (Factor 2), and one group of variables described oral parameters (Factor 1). The loading for the MDEQ was highest in the factor mainly describing oral parameters. One of the questions included in the MDEQ *(“Do you experience dryness of the nose, mouth, throat, chest, or vagina?”)* may be the reason for this overlap. To our knowledge, utilizing factor analysis to explore the relationship between dry eyes and dry mouth is rare. Caffery et al. investigated clinical characteristics (health history, blood analysis, symptoms of dry eye and dry mouth, salivary flow, salivary gland biopsy, tear flow, ocular staining) using factor analysis in a group of patients with primary Sjögren’s syndrome, but could not reveal a dry mouth factor^54^. Thus, our findings are novel, and may serve as a rationale for increased interdisciplinary cooperation between the medical and dental fields.

A limitation of the present study was the male to female ratio. In this study cohort, 45% were men as opposed to 51% in the larger cohort from which this group of participants was recruited. This percentage is also lower than the sex ratio in the general population in Oslo for this age group^55^. The ethnicity and education level were comparable in the two cohorts; however, they were somewhat skewed compared to the general population in Oslo. This skewness is a common finding between responders and non-responders in cross-sectional studies^56,57^. As for the recruitment process, all subjects were primarily invited to the oral health examination prior to enrollment in the current study investigating ocular health. One might argue that a different cohort of participants would have accepted to participate in the study if the larger project’s primary focus was on ocular health.

## Conclusion

In the general population of the young elderly there was a significant, but weak correlation between dry eyes and dry mouth. The participants with more severe dry eye symptoms had worse subjective and objective findings of dry mouth. In this group of young elderly, there was also a positive association between the number of drugs used and the presence of ocular and oral symptoms. Whether this is caused by a qualitative change in tears and saliva remains to be explored in future follow-up studies. The presence of significantly more severe ocular and oral symptoms and oral objective findings in the participants with current or previous systemic diseases calls for increased awareness and an interdisciplinary approach.

## Supplementary Information

Supplementary Table 1.

## Data Availability

The data that support the findings of this study are available from the corresponding author upon reasonable request.
