# Supplementary material for: The relationship between ocular and oral dryness in a cohort from the 65-year-old population in Norway
Source: Sci Rep. 2022 Jun 13;12:9805. doi: 10.1038/s41598-022-13985-6 (PMC9191758; doi:10.1038/s41598-022-13985-6)
Supplement: Supplementary file 1 — Supplementary Table 1. [file 41598_2022_13985_MOESM1_ESM.docx]

| ATC-code | Generic name | n | Potential xerogenic drug |
| --- | --- | --- | --- |
| C10AA05 | [atorvastatin](https://www.whocc.no/atc_ddd_index/?code=C10AA05) | 20 | No |
| H03AA01 | [levothyroxine sodium](https://www.whocc.no/atc_ddd_index/?code=H03AA01) | 17 | No |
| B01AC06 | [acetylsalicylic acid](https://www.whocc.no/atc_ddd_index/?code=B01AC06) | 14 | No |
| C09CA06 | [candesartan](https://www.whocc.no/atc_ddd_index/?code=C09CA06) | 12 | No |
| C10AA01 | [simvastatin](https://www.whocc.no/atc_ddd_index/?code=C10AA01) | 12 | No |
| C07AB02 | [metoprolol](https://www.whocc.no/atc_ddd_index/?code=C07AB02) | 10 | Yes |
| C09CA04 | [irbesartan](https://www.whocc.no/atc_ddd_index/?code=C09CA04) | 8 | No |
| A12AX | [calcium, combinations with vitamin D and/or other drugs](https://www.whocc.no/atc_ddd_index/?code=A12AX&showdescription=no) | 8 | No |
| G03CA03 | [estradiol](https://www.whocc.no/atc_ddd_index/?code=G03CA03) | 8 | No |
| C08CA01 | [amlodipine](https://www.whocc.no/atc_ddd_index/?code=C08CA01) | 7 | No |
| M04AA01 | [allopurinol](https://www.whocc.no/atc_ddd_index/?code=M04AA01) | 6 | No |
| R06AE07 | [cetirizine](https://www.whocc.no/atc_ddd_index/?code=R06AE07) | 6 | Yes |
| C09CA01 | [losartan](https://www.whocc.no/atc_ddd_index/?code=C09CA01) | 6 | No |
| H02AB06 | [prednisolone](https://www.whocc.no/atc_ddd_index/?code=H02AB06) | 6 | No |
| A02BC02 | [pantoprazole](https://www.whocc.no/atc_ddd_index/?code=A02BC02) | 6 | Yes |
| R06AX27 | [desloratadine](https://www.whocc.no/atc_ddd_index/?code=R06AX27) | 5 | Yes |
| B01AC04 | [clopidogrel](https://www.whocc.no/atc_ddd_index/?code=B01AC04) | 5 | No |
| S01XA20 | [artificial tears and other indifferent preparations](https://www.whocc.no/atc_ddd_index/?code=S01XA20) | 5 | No |
| S01ED51 | [timolol, combinations](https://www.whocc.no/atc_ddd_index/?code=S01ED51) | 4 | Yes |
| B01AA03 | [warfarin](https://www.whocc.no/atc_ddd_index/?code=B01AA03) | 4 | No |
| A10BA02 | [metformin](https://www.whocc.no/atc_ddd_index/?code=A10BA02) | 4 | No |
| N02BE01 | [paracetamol](https://www.whocc.no/atc_ddd_index/?code=N02BE01) | 4 | No |
| C09DA06 | [candesartan and diuretics](https://www.whocc.no/atc_ddd_index/?code=C09DA06) | 3 | Yes |
| C09DA01 | [losartan and diuretics](https://www.whocc.no/atc_ddd_index/?code=C09DA01) | 3 | No |
| B01AF02 | [apixaban](https://www.whocc.no/atc_ddd_index/?code=B01AF02) | 3 | No |
| C09DB01 | [valsartan and amlodipine](https://www.whocc.no/atc_ddd_index/?code=C09DB01) | 3 | No |
| C03CA01 | [furosemide](https://www.whocc.no/atc_ddd_index/?code=C03CA01) | 3 | Yes |
| M01AE01 | [ibuprofen](https://www.whocc.no/atc_ddd_index/?code=M01AE01) | 3 | Yes |
| N03AX09 | [lamotrigine](https://www.whocc.no/atc_ddd_index/?code=N03AX09) | 3 | Yes |
| R06AX13 | [loratadine](https://www.whocc.no/atc_ddd_index/?code=R06AX13) | 3 | Yes |
| R03AK07 | [formoterol and budesonide](https://www.whocc.no/atc_ddd_index/?code=R03AK07) | 3 | No |
| A11EA | [vitamin B-complex, plain](https://www.whocc.no/atc_ddd_index/?code=A11EA&showdescription=no) | 3 | No |
| N06AB06 | [sertraline](https://www.whocc.no/atc_ddd_index/?code=N06AB06) | 3 | Yes |
| G03FA01 | [norethisterone and estrogen](https://www.whocc.no/atc_ddd_index/?code=G03FA01) | 2 | No |
| M05BA04 | [alendronic acid](https://www.whocc.no/atc_ddd_index/?code=M05BA04) | 2 | No |
| M01AH05 | [etoricoxib](https://www.whocc.no/atc_ddd_index/?code=M01AH05) | 2 | Yes |
| B03BA01 | [cyanocobalamin](https://www.whocc.no/atc_ddd_index/?code=B03BA01) | 2 | No |
| G04BD12 | [mirabegron](https://www.whocc.no/atc_ddd_index/?code=G04BD12) | 2 | No |
| D07AD01 | [clobetasol](https://www.whocc.no/atc_ddd_index/?code=D07AD01) | 2 | No |
| A02BC05 | [esomeprazole](https://www.whocc.no/atc_ddd_index/?code=A02BC05) | 2 | Yes |
| C10AX09 | [ezetimibe](https://www.whocc.no/atc_ddd_index/?code=C10AX09) | 2 | No |
| N05CF01 | [zopiclone](https://www.whocc.no/atc_ddd_index/?code=N05CF01) | 2 | Yes |
| A10AC01 | [insulin (human)](https://www.whocc.no/atc_ddd_index/?code=A10AC01) | 2 | No |
| C08DA01 | [verapamil](https://www.whocc.no/atc_ddd_index/?code=C08DA01) | 2 | No |
| S01GX02 | [levocabastine](https://www.whocc.no/atc_ddd_index/?code=S01GX02) | 2 | No |
| S01EE01 | [latanoprost](https://www.whocc.no/atc_ddd_index/?code=S01EE01) | 2 | No |
| N03AX12 | [gabapentin](https://www.whocc.no/atc_ddd_index/?code=N03AX12) | 2 | No |
| G03CA04 | [estriol](https://www.whocc.no/atc_ddd_index/?code=G03CA04) | 2 | No |
| C07AA05 | [propranolol](https://www.whocc.no/atc_ddd_index/?code=C07AA05) | 2 | Yes |
| C10AA03 | [pravastatin](https://www.whocc.no/atc_ddd_index/?code=C10AA03) | 2 | No |
| R03AK06 | [salmeterol and fluticasone](https://www.whocc.no/atc_ddd_index/?code=R03AK06) | 2 | Yes |
| N02AX02 | [tramadol](https://www.whocc.no/atc_ddd_index/?code=N02AX02) | 2 | Yes |
| R03AC02 | [salbutamol](https://www.whocc.no/atc_ddd_index/?code=R03AC02) | 2 | Yes |
| A10BJ02 | [liraglutide](https://www.whocc.no/atc_ddd_index/?code=A10BJ02) | 2 | No |
| M01AE52 | [naproxen and esomeprazole](https://www.whocc.no/atc_ddd_index/?code=M01AE52) | 2 | Yes |
| M05BA08 | [zoledronic acid](https://www.whocc.no/atc_ddd_index/?code=M05BA08) | 1 | No |
| L04AA13 | [leflunomide](https://www.whocc.no/atc_ddd_index/?code=L04AA13) | 1 | No |
| C07AB03 | [atenolol](https://www.whocc.no/atc_ddd_index/?code=C07AB03) | 1 | Yes |
| R01AD12 | [fluticasone furoate](https://www.whocc.no/atc_ddd_index/?code=R01AD12) | 1 | No |
| R03BA01 | [beclometasone](https://www.whocc.no/atc_ddd_index/?code=R03BA01) | 1 | No |
| B06AC01 | [c1-inhibitor, plasma derived](https://www.whocc.no/atc_ddd_index/?code=B06AC01) | 1 | No |
| C07AB07 | [bisoprolol](https://www.whocc.no/atc_ddd_index/?code=C07AB07) | 1 | Yes |
| C03CA02 | [bumetanide](https://www.whocc.no/atc_ddd_index/?code=C03CA02) | 1 | Yes |
| C02CA04 | [doxazosin](https://www.whocc.no/atc_ddd_index/?code=C02CA04) | 1 | Yes |
| L04AA06 | [mycophenolic acid](https://www.whocc.no/atc_ddd_index/?code=L04AA06) | 1 | No |
| C03AB01 | [bendroflumethiazide and potassium](https://www.whocc.no/atc_ddd_index/?code=C03AB01) | 1 | Yes |
| N06AB10 | [escitalopram](https://www.whocc.no/atc_ddd_index/?code=N06AB10) | 1 | Yes |
| N06AB04 | [citalopram](https://www.whocc.no/atc_ddd_index/?code=N06AB04) | 1 | Yes |
| C09DA04 | [irbesartan and diuretics](https://www.whocc.no/atc_ddd_index/?code=C09DA04) | 1 | No |
| C10AA07 | [rosuvastatin](https://www.whocc.no/atc_ddd_index/?code=C10AA07) | 1 | No |
| D01AC20 | [imidazoles/triazoles in combination with corticosteroids](https://www.whocc.no/atc_ddd_index/?code=D01AC20) | 1 | No |
| N06DA02 | [donepezil](https://www.whocc.no/atc_ddd_index/?code=N06DA02) | 1 | No |
| G04CA52 | [tamsulosin and dutasteride](https://www.whocc.no/atc_ddd_index/?code=G04CA52) | 1 | Yes |
| R01AD58 | [fluticasone, combinations](https://www.whocc.no/atc_ddd_index/?code=R01AD58) | 1 | Yes |
| N06AX16 | [venlafaxine](https://www.whocc.no/atc_ddd_index/?code=N06AX16) | 1 | Yes |
| C03DA04 | [eplerenone](https://www.whocc.no/atc_ddd_index/?code=C03DA04) | 1 | No |
| H03AA03 | [combinations of levothyroxine and liothyronine](https://www.whocc.no/atc_ddd_index/?code=H03AA03) | 1 | No |
| A10BD08 | [metformin and vildagliptin](https://www.whocc.no/atc_ddd_index/?code=A10BD08) | 1 | No |
| G04CB01 | [finasteride](https://www.whocc.no/atc_ddd_index/?code=G04CB01) | 1 | No |
| B06AC02 | [icatibant](https://www.whocc.no/atc_ddd_index/?code=B06AC02) | 1 | No |
| A10BK01 | [dapagliflozin](https://www.whocc.no/atc_ddd_index/?code=A10BK01) | 1 | No |
| J01XX05 | [methenamine](https://www.whocc.no/atc_ddd_index/?code=J01XX05) | 1 | No |
| S01XA18 | [ciclosporin](https://www.whocc.no/atc_ddd_index/?code=S01XA18) | 1 | No |
| A10BK03 | [empagliflozin](https://www.whocc.no/atc_ddd_index/?code=A10BK03) | 1 | No |
| A10BD11 | [metformin and linagliptin](https://www.whocc.no/atc_ddd_index/?code=A10BD11) | 1 | No |
| D01BA02 | [terbinafine](https://www.whocc.no/atc_ddd_index/?code=D01BA02) | 1 | No |
| H02AB08 | [triamcinolone](https://www.whocc.no/atc_ddd_index/?code=H02AB08) | 1 | No |
| C08CA13 | [lercanidipine](https://www.whocc.no/atc_ddd_index/?code=C08CA13) | 1 | No |
| H03AA02 | [liothyronine sodium](https://www.whocc.no/atc_ddd_index/?code=H03AA02) | 1 | No |
| C07BB07 | [bisoprolol and thiazides](https://www.whocc.no/atc_ddd_index/?code=C07BB07) | 1 | No |
| N03AX16 | [pregabalin](https://www.whocc.no/atc_ddd_index/?code=N03AX16) | 1 | No |
| N04BA02 | [levodopa and decarboxylase inhibitor](https://www.whocc.no/atc_ddd_index/?code=N04BA02) | 1 | Yes |
| N05CH01 | [melatonin](https://www.whocc.no/atc_ddd_index/?code=N05CH01) | 1 | Yes |
| G01AF01 | [metronidazole](https://www.whocc.no/atc_ddd_index/?code=G01AF01) | 1 | No |
| H05BX01 | [cinacalcet](https://www.whocc.no/atc_ddd_index/?code=H05BX01) | 1 | No |
| C01DA14 | [isosorbide mononitrate](https://www.whocc.no/atc_ddd_index/?code=C01DA14) | 1 | No |
| C01BD07 | [dronedarone](https://www.whocc.no/atc_ddd_index/?code=C01BD07) | 1 | No |
| H03BB01 | [carbimazole](https://www.whocc.no/atc_ddd_index/?code=H03BB01) | 1 | No |
| D05BB02 | [acitretin](https://www.whocc.no/atc_ddd_index/?code=D05BB02) | 1 | Yes |
| C08CA05 | [nifedipine](https://www.whocc.no/atc_ddd_index/?code=C08CA05) | 1 | No |
| B03AA01 | [ferrous glycine sulfate](https://www.whocc.no/atc_ddd_index/?code=B03AA01) | 1 | No |
| C01DA02 | [glyceryl trinitrate](https://www.whocc.no/atc_ddd_index/?code=C01DA02) | 1 | No |
| N03AG01 | [valproic acid](https://www.whocc.no/atc_ddd_index/?code=N03AG01) | 1 | No |
| N02AJ06 | [codeine and paracetamol](https://www.whocc.no/atc_ddd_index/?code=N02AJ06) | 1 | Yes |
| R06AD02 | [promethazine](https://www.whocc.no/atc_ddd_index/?code=R06AD02) | 1 | Yes |
| C08CA02 | [felodipine](https://www.whocc.no/atc_ddd_index/?code=C08CA02) | 1 | No |
| L04AD02 | [tacrolimus](https://www.whocc.no/atc_ddd_index/?code=L04AD02) | 1 | No |
| M05BX04 | [denosumab](https://www.whocc.no/atc_ddd_index/?code=M05BX04) | 1 | No |
| C09AA05 | [ramipril](https://www.whocc.no/atc_ddd_index/?code=C09AA05) | 1 | Yes |
| N04BD02 | [rasagiline](https://www.whocc.no/atc_ddd_index/?code=N04BD02) | 1 | No |
| R03AK10 | [vilanterol and fluticasone furoate](https://www.whocc.no/atc_ddd_index/?code=R03AK10) | 1 | No |
| R03BB06 | [glycopyrronium bromide](https://www.whocc.no/atc_ddd_index/?code=R03BB06) | 1 | Yes |
| R03DC03 | [montelukast](https://www.whocc.no/atc_ddd_index/?code=R03DC03) | 1 | No |
| C03DA01 | [spironolactone](https://www.whocc.no/atc_ddd_index/?code=C03DA01) | 1 | Yes |
| N02AX06 | [tapentadol](https://www.whocc.no/atc_ddd_index/?code=N02AX06) | 1 | Yes |
| N03AF01 | [carbamazepine](https://www.whocc.no/atc_ddd_index/?code=N03AF01) | 1 | Yes |
| R06AX26 | [fexofenadine](https://www.whocc.no/atc_ddd_index/?code=R06AX26) | 1 | Yes |
| N06AX03 | [mianserin](https://www.whocc.no/atc_ddd_index/?code=N06AX03) | 1 | Yes |
| N03AX11 | [topiramate](https://www.whocc.no/atc_ddd_index/?code=N03AX11) | 1 | Yes |
| G04BD11 | [fesoterodine](https://www.whocc.no/atc_ddd_index/?code=G04BD11) | 1 | Yes |
| R06AD01 | [alimemazine](https://www.whocc.no/atc_ddd_index/?code=R06AD01) | 1 | Yes |
| C09CA03 | [valsartan](https://www.whocc.no/atc_ddd_index/?code=C09CA03) | 1 | No |
| J05AB11 | [valaciclovir](https://www.whocc.no/atc_ddd_index/?code=J05AB11) | 1 | No |
| M01AB05 | [diclofenac](https://www.whocc.no/atc_ddd_index/?code=M01AB05) | 1 | Yes |
| L02BB04 | [enzalutamide](https://www.whocc.no/atc_ddd_index/?code=L02BB04) | 1 | No |
| S01GX08 | [ketotifen](https://www.whocc.no/atc_ddd_index/?code=S01GX08) | 1 | Yes |
| L02AE03 | [goserelin](https://www.whocc.no/atc_ddd_index/?code=L02AE03) | 1 | No |

***Supplementary Table 1:*** *Overview of all drugs taken by the participants of the study: generic names of the drugs, ATC-codes, number of participants taking the drug, and potential xerogenic effect.*
